# The American Year-Book of Medicine and Surgery

**Published:** 1896-06

**Authors:** 


**The American Year-Book of Medicine and Surgery.** London: The Rebman Publishing Co. (Ltd.) Philadelphia: W. B. Saunders. 1896.

This volume, of 1183 pages, is described as a yearly digest of scientific progress and authoritative opinion in all branches of medicine and surgery, drawn from journals, monographs, and text-books of the leading American and foreign authors and investigators. The matter has been collected and arranged, with critical editorial comments, by twenty-seven editorial colleagues under the general editorial charge of Dr. George M. Gould. It is a handsome volume, issued in most modern style, with blue cloth covers and red edges, profusely illustrated with numerous woodcuts in the text, and thirty-three handsome half-tone and coloured plates, and is the first of a series likely to be known as "Gould's Year-Book'" and its general design is to give in a compact form an "annual summary of medical progress."

It is confidently believed that no very significant fact has escaped review, and the task of condensation into one volume has been a very onerous one. More than half of the editorial staff are leaders of the profession in Philadelphia, many of them having already a world-wide reputation; four are well-known specialists in New York, and four in Chicago, two in St. Louis, two in Cleveland, and one in Columbus, Ohio.

It will be obvious that a book built up in this way deserves more than a casual inspection, and the further one goes into the contents the more is one convinced of the care with which the work has been compiled. The special training and experience of the staff has enabled them to call forth a great degree of judicial criticism, which prevents the reader from being bewildered by a heterogeneous mass of abstracts recording little real advance in knowledge or improvement in treatment.

In a complex work like this there cannot fail to be inconsistencies. For instance, we notice (page 66) that, alluding to Dr. Pavy's new views on the carbo-hydrates, "throughout his lectures there is the evidence of careful and painstaking work, and it is not unlikely that future investigations will show the soundness of Pavy's observations," whereas we find (page 1093), in allusion to Dr. Noël Paton's criticism, that "the resume [*sic*] of the evidence in favor of the orthodox view of the constant production of sugar in the liver and its disappearance in the circulation is compact and convincing."

A three-column index of 55 pages is a necessary part of the volume, and the references are throughout at the bottom of the pages rather than collected at the end. We notice very few errors or inaccuracies, but many innovations in spelling have been made by the editor. Some of these we should like to correct, but they are made "in the interest of much-needed brevity and simplicity."

We heartily congratulate the editor on the volume for 1896, which it will be difficult to surpass.

